# Traumatic Experiences and Mental Health Risk for Refugees

**DOI:** 10.3390/ijerph17061943

**Published:** 2020-03-16

**Authors:** Victoria A. Schlaudt, Rahel Bosson, Monnica T. Williams, Benjamin German, Lisa M. Hooper, Virginia Frazier, Ruth Carrico, Julio Ramirez

**Affiliations:** 1 Department of Psychology, University of Miami, Coral Gables, FL 33146, USA; victoria.schlaudt@miami.edu; 2 Global Health Center, Division of Infectious Diseases, School of Medicine, University of Louisville, Louisville, KY 40292, USA; RBOSSON@partners.org (R.B.); benjamin.german@louisville.edu (B.G.); ruth.carrico@louisville.edu (R.C.); jarami01@exchange.louisville.edu (J.R.); 3 School of Psychology, University of Ottawa, 136 Jean-Jacques Lussier, Vanier Hall, Ottawa, ON K1N 6N5, Canada; 4 Center for Educational Transformation, University of Northern Iowa, Cedar Falls, IA 50614, USA; lisa.hooper@uni.edu; 5 Department of Psychology, Spalding University, Louisville, KY 40203, USA; virginiasfrazier@gmail.com

**Keywords:** Refugees, mental health, violence, trauma

## Abstract

Refugees who settle in Western countries exhibit a high rate of mental health issues, which are often related to experiences throughout the pre-displacement, displacement, and post-displacement processes. Early detection of mental health symptoms could increase positive outcomes in this vulnerable population. The rates and predictors of positive screenings for mental health symptoms were examined among a large sample of refugees, individuals with special immigrant visas, and parolees/entrants (*N* = 8149) from diverse nationalities. Logistic regression analyses were used to determine if demographic factors and witnessing/experiencing violence predicted positive screenings. On a smaller subset of the sample, we calculated referral acceptance rate by country of origin. Refugees from Syria, Iraq, and Afghanistan were most likely to exhibit a positive screening for mental health symptoms. Refugees from Sudan, Iraq, and Syria reported the highest rate of experiencing violence, whereas those from Iraq, Sudan, and the Democratic Republic of Congo reported the highest rate of witnessing violence. Both witnessing and experiencing violence predicted positive Refugee Health Screener-15 (RHS-15) scores. Further, higher age and female gender predicted positive RHS-15 scores, though neither demographic variable was correlated with accepting a referral for mental health services. The findings from this study can help to identify characteristics that may be associated with risk for mental health symptoms among a refugee population.

## 1. Background

### 1.1. Traumatic Experiences and Mental Health Risks for Refugees

As of 2017, there was an estimate of 22.5 million refugees worldwide [1]. Each year the United States government determines the number of refugees who will be permitted to enter the country, with numbers ranging from less than 30,000 to over 200,000 annually [2]. Many of these individuals experience high rates of mental health difficulties in response to potentially traumatic pre-displacement, displacement, and post-displacement events, among other factors such as acculturative stress in a new country [3]. As a result of these lived experiences and events, post-traumatic stress disorder (PTSD), depression, and anxiety are the most prevalent mental health conditions among refugee populations [3]. Unfortunately, many of these mental health symptoms go undiagnosed and untreated due to language and cultural barriers for refugee populations [4]. 

### 1.2. Identifying Refugees in Need of Mental Health Services

Many refugees enter the country without screening or with only informal screenings [4] and may have unidentified pre-displacement or in-transit trauma and trauma sequelae that persist and exacerbate post-displacement struggles. Early intervention of psychiatric concerns in refugees is critical [5]. The Refugee Health Screener-15 (RHS-15) [6] was designed to identify refugees who present with issues that likely demand psychiatric attention. The RHS-15 was standardized to create “diagnostic proxies” for PTSD, depression, and anxiety, with good sensitivity and specificity across diverse nationalities [6,7,8]. Diagnostic proxies were used in place of diagnostic interviews to decrease financial and time resources spent by completing fully structured diagnostic interviews [6]. The RHS-15 is considered an efficient diagnostic instrument for identification of refugees at risk [6]. 

Although the RHS-15 is a promising instrument to detect commonly reported mental health conditions among diverse refugees, challenges in identifying refugees who are in need of mental health care still exist [9]. The ability to identify the groups of refugees who are at the highest risk of developing mental health difficulties allows care providers such as social workers, physicians, and refugee resettlement agencies to direct resources to the refugees with the greatest and most urgent needs. In addition, this knowledge can facilitate organization of community leaders and agencies around important culturally-relevant topics such as the best ways to provide psychoeducation and reducing the stigma associated with mental health and help-seeking behavior.

### 1.3. Risk Factors for Mental Health Symptoms and Barriers to Treatment Services among Refugees 

Previous research has identified trauma as a risk factor for depression (e.g., [10,11,12]), PTSD [11,13], and anxiety [14,15] in refugee populations. A large meta-analysis of the factors associated with depression and PTSD among refugees found that experiencing torture was a main predictor along with experiencing other potentially traumatic events [16]. In addition, Momartin and colleagues (2004) [17] noted that within a sample of Bosnian refugees, several types of trauma (e.g., threat to one’s own life, witnessing a family member’s death, or other events) were associated with co-morbid PTSD and depression, whereas only threat to one’s own life was related to PTSD (without co-morbidities of anxiety or depression). Therefore, there is some evidence that different types of trauma may be related to distinct mental health outcomes. 

Previous research has indicated that both female sex [14,18] and older age [11,18] have been linked with poorer psychological health in refugees, though there are some studies that demonstrate no effect of sex on outcomes [19]. Further research is warranted to clarify which demographic factors are the most predictive of mental health difficulties in refugee populations to help focus and culturally-tailor prevention and intervention efforts.

In addition to the risk factors that may be present, ensuring refugees receive the mental health services they need can be challenging. Often, refugees face barriers such as stigma [20], low access to or quality of services [21], low cultural competence of practitioners [22], or a lack of knowledge about how services work and how they can be helpful [21]. Data on refugee engagement with mental health referrals varies, with studies reporting low referral acceptance (37%; [23]) and others reporting greater success (e.g., 74%; [6] and 50%; [24]). Refugees often turn to spiritual healers or trusted community members to aid them with mental health difficulties instead of mental health professionals [23]. Due to the difficulty in providing mental health services to refugees, it is important to understand factors that may help identify individuals at risk.

Cultural differences may exist in factors related to mental health outcomes and referral acceptance. Three previous studies shed preliminary light on mental health and treatment engagement by country of origin. One study described that 50.5% of Iraqi refugees (47/93), 28% of Bhutanese refugees (21/75), and 10.8% of Burmese (Myanmarese) refugees (9/83) screened positive for mental health concerns on the RHS-15 [6]. Another study examined 112 women seeking obstetrics/gynecological care; 23% (*n* = 26) screened positive for mental health concerns on the RHS-15. Women from “other” Middle East/Asian countries exhibited the highest rate of positive results (3/6 or 50%), followed by Iraqis (14/30 or 46.66%), “other” African nations (5/15 or 33.33%), Somalis (3/22 or 13.63%), and Burmese (1/33 or 3.03%) [25]. In addition, a pilot study was completed in a primary care setting on a sample of 178 refugees from various nations [24]. The results of this study indicate that over half (25/46 or 54.3%) of the Iraqis who were screened exhibited a positive score in this study. Bhutanese refugees exhibited a lower rate of positive screenings (20/114 or 17.5%). Refugees from other nations were screened at a much lower rate (e.g., Somali refugees 2/8, Eritrea 1/1). 

Taken together, this literature demonstrates that Iraqi individuals exhibit high rates of positive mental health screenings, with refugees from other nations demonstrating lower rates (e.g., Bhutanese) or being screened in much lower numbers, which makes it difficult to understand trends in mental health. The current investigation allows for a large-scale view of these studies to bolster and expand previous findings. 

### 1.4. Aims of the Current Study

In order to facilitate rapid identification of vulnerable refugees, the current study aims to determine prevalence of traumatic experiences and positive screenings for symptoms of depression, anxiety, or PTSD by country of origin [6]. In addition, this study will examine the impact of factors such as age, sex, and prior traumatic experiences on mental health outcomes. Knowing risk factors such as trauma experience, demographic factors, and country of origin could be helpful with prevention and screening efforts to address mental health concerns in a timely, productive fashion. 

In addition, the current study aims to identify rates of referral acceptance across nations. This information could help practitioners culturally-tailor their conversations appropriately with those from countries that tend to be reluctant to accept referrals. They could also ensure more frequent mental health screenings during physical health appointments, or work with community leaders and community organizations to find ways to increase referral acceptance rates (e.g., new ways to introduce the referrals). Further, information regarding referral acceptance could inform the type of referrals provided. For example, refugee groups who accept referrals at a low rate may benefit from referral to community-based services or integrated primary and mental health care [9].

## 2. Method

### 2.1. Procedure and Participants

The current study utilizes the Arriving Refugee Informatics Surveillance and Epidemiology database (ARIVE), which comprises of data that were collected from six refugee health screening sites in Kentucky between October 2012 and June 2016. The ARIVE database holds information from the refugee health assessment, which is funded by the Office of Refugee Resettlement (ORR) to utilize within eight months of refugee arrival. Agencies strive to complete the non-mandatory screenings within 90 days of arrival. The process is overseen by the Kentucky Office for Refugees (KOR), which ensures that the screening is standardized and that the clinics that screen offer referrals and use appropriate interpreting services. The clinics that provide the screening must submit their data to the ARIVE database. Participating agencies include a private clinic, urgent care clinics, university clinics, and federally qualified health centers. The full sample includes 8149 individuals with immigration statuses including refugee, entrant/asylee, and special immigrant visa.

This study was approved by the University of Louisville IRB in 2012 (ID #12.0143).

### 2.2. Measures

Demographic data including age, sex, country of origin, and immigration status were collected. In addition, on a subset of the sample, information regarding acceptance of a referral for mental health services was recorded. 

The RHS-15 [6] is a 15-item measure designed to screen for emotional distress predictive of depression, anxiety, and PTSD in refugees being resettled, including 13 symptom items, one item regarding coping, and a distress thermometer. The measure is a Likert-type (0 = not at all, 4 = extremely) scale allowing refugees to endorse the level of difficulty they experience from each of the symptoms presented. Respondents are asked to “indicate the degree to which the symptom has been bothersome to you over the past month.” Options include items such as, “Feeling down, sad, or blue most of the time” and “Nervousness or shakiness inside.” A positive screening is identified by a score of 12 or more and/or a score greater than five on the distress thermometer. This scale has demonstrated good specificity and sensitivity in detecting mental health concerns [6].

Additional questions were asked regarding whether each participant had experienced and witnessed imprisonment, torture, or violence (e.g., “Have you experienced imprisonment, torture, or violence?”; “Have you witnessed someone experiencing torture or violence”). A follow up question about each experience was then asked, with the option to provide more details if they chose (e.g., “have you experienced imprisonment?”; “have you experienced torture?”). 

At the time of this study, the RHS-15 was available in Arabic, Burmese, Karen, Nepali, Somali, Farsi, Russian, French, Amharic, Tigrinya, Swahili, and Spanish, see Pathways to Wellness (2011) [26] for more information regarding the translation process. Interpreters were used for any other languages needed.

### 2.3. Analysis

Due to low sample sizes from some countries, screening data are only reported for countries where *n* > 30. First, frequency of traumatic experience and positive mental health screener were determined by country of origin. Next, logistic regression was utilized to examine which demographic factors and traumatic experiences predicted positive screenings. Additionally, the rate at which refugees accepted referrals if their screening was positive was also investigated. The percentage was calculated based on the number of patients who accepted the referral over the total number of positive screens for a given nationality. Correlations of age and sex with referral acceptance were conducted. Missing cases were deleted.

## 3. Results

### 3.1. Positive Screenings and Frequency of Trauma

A mental health screening was completed with 8149 refugees, with 22.13% endorsing a positive screening (i.e., score of 12 or greater). The nationalities with the highest rates of positive scores were as follows: Syrians (51.22%), Iraqis (49.65%), and Afghanis (39.45%). Refugees from Sudan experienced torture, violence, or imprisonment at the highest rate (23.53%), followed by Iraqis (22.44%), and Syrians (22.37%). Witnessing torture or violence occurred most among Iraqis (57.80%), Sudanese (50%), and individuals from Democratic Republic of Congo (43.42%). See Table 1 for further information. 

### 3.2. Predictors of Positive Screening

A logistic regression analysis was conducted to determine the relative contributions of age, sex, witnessing a traumatic event, and experiencing a traumatic event in predicting a positive screen (see Table 2). Due to missing data, the analysis consisted of 5278 participants. The full model was significant, (χ^2^ (4) = 571.49, *p* < 0.001, Nagelkerke’s R^2^ = 0.156). All four predictors were significantly associated with positive screenings, witnessing trauma (β = 1.09, *p* < 0.001), sex (β = 0.81, *p* < 0.001), age (β = 0.03, *p* < 0.001), and experiencing trauma (β = 0.63, *p* < 0.001). Individuals who witnessed trauma were 2.96 times more likely to exhibit a positive RHS-15 score, and female refugees had increased odds of a positive screening by 2.24 times. Experiencing trauma was associated with a 1.87 increased likelihood of a positive screening, and each year of age was associated with an increase in odds at a rate of 1.03. 

### 3.3. Referral Acceptance

After patients received an initial screening, data were tracked for a subset of the sample regarding referral acceptance (*n* = 237). Those who screened positive were offered a referral for mental health care. Only Cuba and Iraq had sufficient referral data available (*n* > 30). Cuban participants accepted referrals at a higher rate (66.66%) than Iraqi participants (57.81%), but this difference was not significant (χ^2^ = 1.28, *p* = 0.26). However, it is important to note that in other nations (with lower reporting rates), rate of referral acceptance varied greatly, ranging from 10% to 83.3%. Age and sex were not significantly correlated with referral acceptance.

## 4. Discussion

The purpose of the current study was to examine the level of risk of individuals from specific countries experiencing mental health difficulties. Results indicated that the highest rate of positive screenings was among refugees from Syria, Iraq, and Afghanistan, respectively. In addition, experiencing trauma, witnessing trauma, female sex, and older age were all associated with an increased risk for screening positive. Finally, referral acceptance rates varied between 57.81% and 66.66% in Iraqi and Cuban refugees, respectively. Demographic factors were not related to referral acceptance.

This study expands on prior research examining positive mental health screening by country of origin [6,24,25], as it provides information about a larger group of refugees from diverse nations. Interestingly, the rate of positive scores by Iraqi refugees was nearly identical between these three studies (49.65% in current study, between 46.6% [25] and 54.3% [24] in previous studies), which indicates that Iraqi refugees consistently report high levels of mental health distress. Across these studies, refugees from some countries, such as Bhutan, exhibited positive scores at lower rates (20.3% in the current study as compared to 17.5% [24] and 28% [6]). It is vital to attend to the mental health needs of refugees from all nations, but the knowledge that current incoming refugees from Afghanistan, Syria, and Iraq tend to demonstrate higher risk for positive screenings can help focus assessment and prevention efforts. Additionally, the current investigation found that sex and age made a unique contribution to mental health outcomes. This finding is consistent with past studies citing that women and older refugees are at a heightened risk for mental health difficulties [18]. Women may be at higher risk due to increased sexual victimization [27], whereas older refugees may have higher risk due to the greater accumulation of traumas over time [16].

Overall, information regarding country of origin, demographics, and mental health can aid individuals who provide care to refugees in their screening and intervention efforts. For example, it may be useful to prepare additional culturally and linguistically appropriate psychoeducational material regarding mental health refugees from nations that endorse high levels of mental health distress (e.g., Syrians, Iraqis, Afghanis), or organize community leaders around these issues.

### 4.1. Trauma and Positive Screenings

Both witnessing and experiencing trauma/torture are related to positive screenings on the RHS-15. However, those who have witnessed torture may be at higher risk for mental health difficulties than those who have experienced torture themselves. This finding may be related to the concept of survivor’s guilt. Although different disciplines have defined this term in disparate ways, it is viewed as a process by which a person experiences distress following being spared from harmful events that happened to others. The guilt experienced by the survivor is often viewed as a way to sustain a connection to loved ones who suffered or died [28]. The negative consequences of witnessing traumatic events that occur to another may also relate to collectivistic values, which are common in countries in Asia, Latin America, and Africa [29]. Kim (1995) [29] describes that collectivistic cultures “stress ‘we’ consciousness, collective identity, emotional dependence, group solidarity, sharing, duties and obligations, need for stable and predetermined friendship, group decision, and particularism” (p. 4). Refugees from collectivistic societies, therefore, may stress the impact of the trauma on the group, rather than on the individual when witnessing such events, which may help explain this finding. 

Past studies have found that specific risk factors such as poor integration into host community, lack of social and vocational functioning, and family dysfunction are related to less favorable mental health outcomes in refugees [30]. However, there is considerably less research regarding how witnessing versus experiencing traumatic events is related to mental health outcomes. Future studies should continue to clarify these variables and their mechanisms to help create a greater understanding of how mental health professionals can intervene to ameliorate mental health issues among refugees. In addition, this information can be useful to clinicians or agencies working with refugees who have experienced trauma; if their relatives witnessed the trauma, they may benefit from psychiatric referrals as well. This may be particularly relevant for refugees from Iraq, Sudan, and the Democratic Republic of Congo, as refugees from these nations reported the highest rates of witnessing traumatic events. That being said, refugees from all nations may benefit from mental health interventions, regardless of witnessing or experiencing torture, since family members (even those who did not witness or experience the trauma and are second or third generations of survivors) may also experience many mental health issues.

Importantly, research regarding the resilience factors related to refugee mental health and its relation to country of origin is vital. For example, although Sudanese individuals experienced the highest rate of torture and witnessed it the second highest percentage, they did not report the highest rate of positive screenings. Past research has demonstrated that factors such as perceived social support, religious affiliation, separation from parents, coping ability, and poor living conditions all affect resilience [31]. In addition to this information, studies that aim to understand country-specific resilience factors may help refugee agencies, mental health providers, and community leaders find ways to bolster resiliency and decrease mental health distress.

### 4.2. Referral Acceptance

Due to small sample sizes, it was not possible to effectively compare referral acceptance rates by nation, and continued research in this area is vital. Past studies indicate that stigma is often a challenge faced by refugees and is a barrier to mental health treatment [32,33,34,35,36]. Stigma may be experienced from the community, family, or individual. However, the current understanding of the stigma they perceive does not seem to differentiate why refugees from some nations may accept referrals at higher rates. For example, current literature does not detail if a refugee from Iraq will experience more, or qualitatively different, stigma than a refugee from another nation. Previous studies reported referral acceptance rates ranging from 37% to 74% [6,23,24], which indicates that there may be specific factors related to how likely individuals are to seek mental health services when referred. 

Reasons for the disparity in referral acceptance rates are an important area in need of ongoing research. Our current understanding of referral acceptance is limited, which decreases the ability to create interventions that are specific to the various groups that comprise this vulnerable population. It is unclear, for example, if structural barriers to care (e.g., financial issues, transportation, child care) or personal characteristics (e.g., educational level) are the main reason for low referral acceptance rates for specific groups, or if there are other factors that may be more easily manipulated such as psychoeducation and perceived cultural competence of the provider. Stigma has been noted as a barrier by Cuban and Syrian, refugees, and is undoubtedly an important target for intervention [37,38]. Additionally, future research could help to clarify policies and actions to be taken by community leaders, politicians, care providers, and refugee agencies.

As mentioned above, one policy that may increase referral acceptance is to refer refugees for medical health, primary care, and mental health services within the same office [9]. This facilitates follow-up by lowering the burden of navigating different health systems, a new location, and also may reduce the stigma associated with mental health issues and services. Although this solution is helpful, future research regarding the association between referral acceptance and country of origin can guide this intervention and others, as cultural factors may relate to engagement.

### 4.3. Limitations and Future Directions

Although this investigation provides important information about trauma experienced by refugees from select countries of origin, there are several limitations. The RHS-15 [6] as a measure is limited in scope, as it lacks items about thoughts of self-harm, suicidal ideation, or exposure to torture. Subsequent revisions of the instrument should consider the addition of these items to better capture the full range of trauma symptoms often experienced by refugees. Future studies could also consider utilizing culturally adapted screening measures in addition to the RHS-15 to understand benefits to each type of assessment. In addition, the added straightforward questions about experience of trauma may have led to refugees denying symptoms and experiences due to lack of comfort and stigma regarding mental health. Therefore, some individuals may have under-reported their true level of symptomatology and previous traumas. Further, it may be important to find new ways to assess mental health risks in individuals from countries screening positive at a low rate; for example it is possible that Bhutanese individuals endorsed mental health concerns at a lower rate due to the manner of assessment or stigma over revealing mental health problems, rather than a true difference in symptoms.

An additional limitation was the small sample sizes evinced in some subsamples. Due to low sample sizes for most countries of nationality under investigation, we did not calculate acceptance rates of mental health referrals for many patients who screened positive. In addition, this study did not clarify how connected to their country of origin participants felt and how that may relate to referral acceptance and risk. It may be helpful, in future studies, to examine the culture a refugee identifies most with, and how that relates to mental health outcomes and referral acceptance. Overall, more research is needed to understand nationality-specific reasons for declining mental health referrals and ways organizations and service providers can intervene.

Last, this study does not provide data regarding other risk and resilience factors related to refugee mental health outcomes. Our data did not account for other variables related to mental health for refugee individuals, such as the number of traumas experienced, development level of village/city of origin, change in status due to refugee experience, length of time in refugee camp before resettlement, work status and type prior to departure from country of origin, and education level. As we learn more about refugee populations and their unique challenges and resilience, our models of mental health outcomes and needs will become more nuanced. However, despite these limitations, this study can provide important data to clarify information regarding country of origin, trauma, and their association to mental health.

## 5. Conclusions

This article can help to provide information regarding refugees who are at risk for mental health symptoms as it relates to traumatic experiences and demographic information. Refugee agencies and service providers can use this evidence to prepare appropriately for working with these at-risk populations. It is our hope that planning for these unique challenges and risk factors can help to increase access to, and effectiveness of, the mental health services available for refugee individuals.

## Figures and Tables

**Table 1:** Distribution of respondents who witnessed/experienced torture, violence, and imprisonment, recorded Refugee Health Screener-15 (RHS-15) positive, and referral acceptance rate by country of origin.

Country	Witnessed	Experienced	RHS Positive	Referral Acceptance
Afghanistan	22.4% (28/125)	14.20% (24/169)	39.45% (43/109)	-
Bhutan	1.71% (9/526)	2.22% (16/720)	20.33% (110/541)	-
Burundi	-	14.89% (7/47)	-	-
Congo, D.R.	43.42% (188/433)	17.91% (139/776)	25.83% (100/458)	-
Cuba	5% (129/2576)	7.94% (227/2860)	15.98% (416/2604)	66.66% (62/93)
Eritrea	-	10% (4/40)	-	-
Iraq	57.80% (404/699)	22.44% (228/1016)	49.65% (354/713)	57.81% (37/64)
Myanmar	22.42% (124/553)	15.04% (122/811)	16.83% (86/511)	-
Somalia	33.19% (155/467)	13.44% (107/796)	10.50% (50/476)	-
Sudan	50% (38/76)	23.53% (28/119)	35.14% (26/74)	-
Syria	38.64% (34/88)	22.37% (34/152)	51.22% (42/82)	-
Total	20.45% (1164/5687)	12.77% (976/7645)	22.13% (1266/5721)	56.96% (135/237)

*Note.* Total reported in table includes only those groups with >30 members. Numbers in parentheses indicate the number of individuals who answered yes/screened positive/accepted referral over the total number of individuals possible. Missing cases were deleted. Total sample size is *N* = 8149.

**Table 2:** Logistic Regression for RHS-15 positive scores.

Predictor	β	SE	df	*p-Value*	Exp(B)
Age	0.03	0.003	1	<0.001	1.03
Gender	0.81	0.07	1	<0.001	2.24
Witness	1.09	0.09	1	<0.001	2.96
Experience	0.63	0.10	1	<0.001	1.87
Constant	−3.02	0.12	1	<0.001	0.05
Full Model χ^2^ (4) = 571.49, *p* < 0.001, *n* = 5278					

Experience = experienced torture, imprisonment, or violence. Witness = witnessed torture, imprisonment, or violence; β = beta weight, SE = standard error, df = degrees of freedom.
